# Nanoencapsulation of Mandarin Essential Oil: Fabrication, Characterization, and Storage Stability

**DOI:** 10.3390/foods11010054

**Published:** 2021-12-27

**Authors:** Amer Ali Mahdi, Qais Ali Al-Maqtari, Jalaleldeen Khaleel Mohammed, Waleed Al-Ansi, Sahibzada Muhammad Aqeel, Haiying Cui, Lin Lin

**Affiliations:** 1 Department of Food Quality and Safety, School of Food and Biological Engineering, Jiangsu University, Zhenjiang 212013, China; amer.alimahdi@yahoo.com (A.A.M.); linl@ujs.edu.cn (L.L.); 2 Department of Food Science and Nutrition, Faculty of Agriculture, Food and Environment, Sana’a University, Sana’a 12544, Yemen; qaisalialmaqtari@yahoo.com (Q.A.A.-M.); ansiwaleed@yahoo.com (W.A.-A.); 3 State Key Laboratory of Food Science and Technology, School of Food Science and Technology, Jiangnan University, Wuxi 214122, China; jalal192007@yahoo.com; 4 School of Biotechnology, Jiangnan University, Wuxi 214122, China; aqe302@hotmail.com; 5 State Key Laboratory of Utilization of Woody Oil Resource, Hunan Academy of Forestry, Changsha 410007, China

**Keywords:** *Citrus reticulata*, nanoencapsulation, encapsulation wall materials, molecular docking

## Abstract

This study evaluates the combined efficiency of whey protein isolate (WPI) with maltodextrin (MD) and gum arabic (GA), as a delivery system for encapsulating *Citrus reticulata* essential oil (CEO). The wall materials blended at different rates were produced to obtain seven formulations of nanocapsules (NCEO), namely NCEO-GA, NCEO-MD, NCEO-WPI, NCEO-GA/MD, NCEO-GA/WPI, NCEO-MD/WPI, and NCEO-GA/MD/WPI. The interaction between CEO and WPI was simulated by molecular docking. Findings showed that the physicochemical characteristics and storage stability of formulations containing WPI were considerably improved. The NCEO-GA/MD/WPI formulation demonstrated the optimum values of encapsulation efficiency (92.08%), highest glass transition temperature (79.11 °C), high crystallinity (45.58%), high thermal stability (mass loss at 100 °C < 5%), and also had the highest antioxidant activity and lowest peroxide value after storage. This study demonstrated that combining WPI with MD and GA, as wall material encapsulation, can produce nanocapsules with superior properties to those created using polysaccharides individually.

## 1. Introduction

*Citrus* (Rutaceae, Aurantioideae, Citrinae) is a diverse genus that includes many species. Mandarin (*C. reticulata* Blanco) is one of the species of *Citrus* [1]. The essential oils (EOs) of mandarin have a high content of active compounds, including phenolic, antioxidants, and antibacterial compounds [2,3]. However, using plant-derived bioactive compounds in food products has several limitations, including their sensitivity to light, enzymes, temperature, oxygen, low stability, and pH [4]. Therefore, nutraceuticals and food industries have started adopting new techniques that utilize natural biopolymer systems to deliver the natural bioactive compounds and protect them during treatment and storage [5].

Encapsulation is a viable option for improving the use of active compounds in food products or protecting them from degrading environmental conditions [6]. Encapsulation is described as a carrier matrix in which a bioactive material is encapsulated, allowing the control of the bioactive release rate [7]. Therefore, nanoencapsulation is an efficient strategy for increasing the stability of bioactive compounds, protecting them from interactions with food elements and improving their bioactivity [8]. This strategy also protects bioactive composites from heat, pressure, oxygen, or light [9,10]. Nanoencapsulation is defined as the use of a carrier with a diameter of less than 1 micron (1000 nm) and has characteristics distinct from conventional encapsulation [11].

In addition to encapsulation techniques, the type of wall material used for encapsulation also plays a considerable role in nanocapsules properties [12]. Proteins and polysaccharides have been commonly used in encapsulation [13,14]. However, a single wall material does not satisfy the need for high powder recovery while maintaining acceptable quality [15]. As a result of their combined properties, such as electrostatic repulsion and viscosity increase, the integration of proteins with polysaccharides in the encapsulation process is widely considered as successful in stabilizing emulsions [16].

However, several published works mentioned that whey protein isolate (WPI), maltodextrin (MD), and gum arabic (GA) are considered typical wall materials for encapsulation [17,18,19,20], where MD has good characteristics, such as low viscosity at high concentrations, film-forming capability, and high solubility [21,22]. MD is a weak emulsifier, despite its widespread use in the food industry. Moreover, mixing MD with an efficient emulsifier is recommended to improve its characteristics. GA is also an effective agent in encapsulation procedures because of its colloid functionality. GA is compatible with most starches, gums, proteins, and carbohydrates, and it forms the most stable emulsions with oils across a wide pH range [23].

Nonetheless, the price of GA increases due to its rising demand; therefore, researchers seek to find its suitable alternative or replacement with different proportions [24]. WPI is one of the best coating agents for oils and volatile compounds due to hydrophilic and hydrophobic amino acids, which aid in the encapsulation of hydrophobic substances [19]. Moreover, WPI, a dairy industry by-product, is reported to help stabilize emulsions by lowering the interfacial tension and creating strong competitive interactions. The significance of WPI in emulsion viscosity stabilization and flow behavior modulation has been documented [25]. Moreover, WPI is a functional protein with unique drying characteristics, such as high yield, shielding, and smooth flow [17].

To our knowledge, no study was carried out to encapsulate the mandarin essential oil using MD, GA, and WPI, individually or in combination, as wall materials. Therefore, this study aims to measure the appropriateness of MD, GA, and WPI with *Citrus reticulata* essential oil (CEO), to produce nanocapsules and determine their roles to improve the physicochemical properties and storage stability of the nanocapsules of *Citrus reticulata* essential oil (NCEO), while also confirming the role of WPI by simulating the interaction with CEO by molecular docking.

## 2. Materials and Methods

### 2.1. Material and Chemicals

CEO was provided by J.E. International (Caussols, France). GA (MW: 406.44 KDa, viscosity: 0.024 Pa.s) was purchased from Sinopharm Chemical Reagent Co., Ltd. (Shanghai, China). MD (MW: 4.34 KDa, viscosity: 0.003 Pa.s), and WPI (MW: 40.64 KDa, viscosity: 0.006 Pa.s) was purchased from Shyuanye Co., Ltd. (Shanghai, China). All chemicals used were of analytical grade.

### 2.2. Methods

#### 2.2.1. Preparation of Emulsion

The CEO was encapsulated with three various types of wall materials, including GA, MD, and WPI. The wall materials were combined at different rates to obtain seven formulations (Table 1). To achieve total hydration, these wall ingredients were dissolved in distilled water and constantly agitated overnight at 25 °C. As a carrier oil, corn oil was mixed with CEO and the emulsifier (Tween 20) in proportions of 3.5:3.5:1 (*w*/*w*/*w*). The oil phase was homogenized at 10,000 rpm for 5 min using a high-speed homogenizer (Ultra-Turrax IKA T18 IKA Works Inc., Wilmington, DE, USA). The nanoemulsions were created by slowly adding the oil phase to the aqueous phase and homogenizing for 6 min at 25,000 rpm.

#### 2.2.2. Freeze-Drying Process

The NCEO was prepared in accordance with Muhoza et al. [26], with some modifications. The CEO/W nanoemulsions were previously frozen at −18 °C for 24 h, then dried at −80 °C for 72 h, at 0.05 Mbar using a freeze dryer (Ningbo Scientz Biotechnology Co. Ltd., Ningbo, China). The dried powders were collected and kept for further analysis.

### 2.3. Properties of Emulsions

The mean size diameter (MSD), polydispersity index (PDI), and zeta potential (ζ-potential) of CEO nanoemulsions and the reconstituted NCEO were estimated using a dynamic light scattering device (Zetasizer Nano ZS^®^, Malvern Instruments Ltd., Malvern, Worcestershire, UK). The reconstituted emulsions were obtained by mixing 1 g of the NCEO into 200 mL of deionized water and mixing them using a magnetic stirrer at 500 rpm, at 25 °C for 30 min [27].

### 2.4. Properties of CEO Nanocapsules

#### 2.4.1. Encapsulation Efficiency

The encapsulation efficiency (EE) of NCEO was calculated as the percentage between the surface and total oils of the nanocapsules. According to Mohammed et al. [28], the surface and total oils were estimated, and the EE of NCEO was calculated by using Equation (1) as follows:

(1) $$\mathrm{EE}\;\left(\%\right)=\frac{\mathrm{Surface}\;\mathrm{oil}\;\mathrm{content}\;-\;\mathrm{Total}\;\mathrm{oil}\;\mathrm{content}\;}{\mathrm{Total}\;\mathrm{oil}\;\mathrm{content}\;}\times 100\;$$

#### 2.4.2. Moisture Content, Hygroscopicity, Solubility, and Wettability

Moisture content was measured by drying the NCEO with hot air in an oven for 24 h at 105 °C, and the result was exhibited as a percentage (%). By contrast, NCEO solubility was measured by adding 1 g of nanocapsules into 25 mL of deionized water and mixed for 2 h at 500 rpm. The tubes were centrifuged, and the supernatant was transferred entirely to a crucible and oven-dried overnight at 105 °C. The solubility values were presented as a percentage (%). NCEO hygroscopicity (%) was determined after the storage of the NCEO for 7 days at 75.29% relative humidity (saturated solution of sodium chloride). NCEO wettability was determined by calculating the time (min) required to immerse 1 g of NCEO on a surface of the distilled water (400 mL) at 25 °C [29].

#### 2.4.3. Bulk, Tapped, and Particle Density

The bulk and tapped densities of NCEO were determined, according to the described method by Saifullah et al. [30]. Particle density was measured following the method of Santhalakshmy et al. [31].

#### 2.4.4. Porosity, Cohesiveness, and Flowability

Based on particle and tapped densities, NCEO porosity was measured following the method of Santhalakshmy et al. [31]. The cohesiveness of the nanocapsules powders was estimated using the Hausner ratio (HR), and the compressibility was estimated using the Carr’s index (CI). At the same time, NCEO flowability was determined based on CI and HR, according to Saifullah et al. [30].

#### 2.4.5. Color

A HunterLab Spectrophotometer (UltraScan PRO, Reston, VA, USA) was used to measure the color parameters (lightness (L*), redness (a*), yellowness (b*), and ΔE*) of NCEO, according to Mahdi et al. [29].

#### 2.4.6. Glass Transition Temperature (Tg)

The glass transition temperatures (Tg) of NCEO formulations were measured via differential scanning calorimetry (Q2000, TA Instruments, New Castle, DE, USA), as reported by Mohammed et al. [28], with slight modifications. Approximately 4 mg of NCEO was heated from −50 °C to 150 °C at 10 °C /min (heating rate) under a nitrogen atmosphere (60 mL/min).

#### 2.4.7. Thermogravimetric Analysis (TGA)

The thermal stability of the NCEO preparations was analyzed based on thermogravimetry (TG) and derivative thermogravimetry (DTG), using a TA Instrument (TGA2, Mettler Toledo Corp., Greifensee, Switzerland), at temperatures ranging from 50 °C to 400 °C, 10 °C/min (heating rate), and 50 mL/min (nitrogen flow rate) [32].

#### 2.4.8. X-ray Diffraction (XRD)

The relative crystallinity of the NCEO was predicted via XRD analysis using a D2 Phaser XRD diffractometer (Bruker AXS Co. Ltd., Karlsruhe, Germany) [29]. The scanning speed was set at 10°/min, and patterns were set in the 2θ region between 5° and 60°. The software (MDI Jade 6) was used to analyze X-ray diffraction patterns.

#### 2.4.9. Molecular Docking Simulation

A docking study was performed using BIOVIA Discovery Studio Visualizer (Dassault Systèmes, Vélizy-Villacoublay, France), as reported by Khalifa et al. [33]. The structure of β-lactoglobulin (ID:4DQ3), as a predominant protein of WPI, was obtained from the RCSB Protein Data Bank (https://www.rcsb.org/structure/4DQ3 (accessed on 18 August 2021), according to Stănciuc et al. [34]. As the major compound present in CEO [35], the 3D structure of D-limonene (PubChem CID: 440917) was obtained from the PubChem database (https://pubchem.ncbi.nlm.nih.gov/compound/440917 (accessed on 22 August 2021), as described by Nidhi et al. [36].

#### 2.4.10. Scanning Electron Microscopy (SEM)

The NCEO’s morphology was investigated using an SEM device (SU 1510, Hitachi Corp., Tokyo, Japan). With double scotch tape, the samples were mounted on a specimen holder, then a thin layer of gold was sprayed onto the samples under a vacuum. In the end, a 5 kV accelerating beam voltage was used to scan the samples [29].

#### 2.4.11. Storage Stability

The NCEO stability was evaluated by storing the nanocapsules at 50 °C and 5 °C for 30 days in sealed glass containers. The NCEO stability was estimated at the end of the storage period considering the antioxidant activity, antioxidant capacity, and oxidative stability. The antioxidant activity of the stored NCEO was measured by DPPH assay, and the results were displayed as DPPH inhibition rate. By contrast, the antioxidant capacity was measured by ABTS assay, and the result was exhibited as mg Trolox/g NCEO, according to Al-Maqtari et al. [27]. Meanwhile, the oxidative stability of stored NCEO was evaluated based on the peroxide value. The result was presented as mM hydroperoxide/g oil according to the standard curve of cumene hydroperoxide, as described by Mohammed et al. [28].

### 2.5. Statistical Analysis

All statistical analyses were performed using IBM SPSS 20 software (SPSS Inc., Chicago, IL, USA). Significant differences between the samples were determined via ANOVA by using Duncan’s test (*p* ≤ 0.05).

## 3. Results

### 3.1. MSD, PDI, and ζ-Potential of Fresh Emulsions

The MSD and PDI are important aspects of the physical and rheological features of colloidal systems, including controlled release, stability, and encapsulation efficiency. The MSD of the emulsions ranged from 357.86 to 674.18 nm. The WPI-based nanoemulsion had the lowest MSD, whereas the GA-based nanoemulsion had the highest MSD (Table 2). Results showed that the presence of WPI in the nanoemulsions reduced the MSD, whereas the presence of GA increased this property.

PDI is typically used to measure particle size distribution in suspension. A low PDI indicates a homogeneous particle size distribution with a uniform desired diameter [37]. The PDI of the samples ranged from 0.17 to 0.26. The WPI- and GA-based nanoemulsions, respectively, had the lowest and highest PDI (Table 2). All nanoemulsions had PDI values less than 0.5, indicating that the droplets were distributed uniformly [38].

Surface charge (zeta potential) is an essential physical characteristic exhibited by particles in emulsions. Moreover, the surface charge is a useful index of interactions among particles in a colloidal system and is extensively used to evaluate emulsion stability. Particles with zeta potentials larger than −30 or 30 mV are considered stable, wherein electrostatic repulsion prevents aggregation among emulsion droplets [39]. Emulsion particles at high zeta potentials have a high predisposition to repel each other. As a result, the particles are prevented from getting too close to each other, decreasing particle aggregation [38]. All emulsions in this study were stable, according to the obtained values of the zeta potential. The zeta potential of the emulsions ranged from −58.08 to −57.48 mV. The GA- and MD-based nanoemulsions had the lowest and highest zeta potentials (Table 2).

### 3.2. Properties of CEO Nanocapsules

#### 3.2.1. Encapsulation Efficiency

The efficiency of encapsulation is one of the most important quality parameters of encapsulated EOs [40]. Table 3 shows that the encapsulation efficiency was significantly different (*p* ≤ 0.05) between the NCEO. Meanwhile, no significant difference (*p* ≤ 0.05) was found between the prepared NCEO based on polysaccharides without WPI, and the encapsulation efficiency of the NCEO varied from 36.29% to 92.08%. However, the encapsulation efficiency of NCEO-GA/MD/WPI was higher (92.08%) than the other preparations, whereas that of the NCEO-MD was the lowest (36.29%), followed by NCEO-GA (37.17%). These results were consistent with those of Mohammed et al. [41] (33.33–92.71%) and Fernandes et al. [40] (48.0–93.0%), and higher than those of Fernandes et al. [42] (26.31–61.81%) and Özbek et al. [43] (62.41–71.04%).

The type and quality of the wall material are some of the most significant variables in the preservation of the core material. Thus, the differences in encapsulation efficiency in NCEO preparations can be related to the wall materials. Therefore, the results show that incorporating WPI into the wall structures improves and raises the encapsulation efficiency compared with polysaccharides. This finding is consistent with what was reported by Mahdi et al. [29], regarding the positive impact on encapsulation quality due to the presence of WPI with GA in formulations. No one wall material provides all the properties required for an optimal encapsulating agent; therefore, a mixture of wall materials can improve the qualities of the powder [12]. The combined emulsification characteristics of WPI and polysaccharides can also be responsible for the maximum encapsulation efficiency. According to Lekshmi et al. [21], the mixture of wall materials, particularly protein–polysaccharides, encapsulates the core material more efficiently than a single wall material due to its combination properties. The researchers, in another study, reported that the increased WPI ratio in the wall matrix resulted in the improved encapsulation efficiency and small particle size of wheat germ oil microcapsules [44].

#### 3.2.2. Particle Size and PDI of Nanocapsules

The particle size and PDI of reconstituted nanocapsules are critical parameters to consider because they are connected to release, stability, and compound distribution [45]. Raeisi et al. [46] confirmed that the capsules with PDI values close to 0.4 are appropriate for the food industry. Table 3 shows that the particle size of reconstituted NCEO ranged from 427.35 nm to 782.09 nm, while the PDI values ranged from 0.26 to 0.47.

The NCEO-WPI formulation had the smallest particle size and PDI. Simultaneously, this formulation was not significantly different compared with the NCEO-MD/WPI formulation. By contrast, the NCEO-GA formulation had the largest particle size and PDI values. The particle size is usually considered in relation to wall material, surfactant, and EO type [47]. Therefore, these results indicated that the WPI decreased the particle size of polysaccharide-based wall materials and PDI compared with a single or combination of polysaccharides. This phenomenon led to the improved characteristics of the nanocapsules produced in this study. Such an improvement was confirmed by Özbek and Ergönül [43], who indicated that increasing the WPI ratio in wall materials led to a decrease in the particle size of wheat germ oil microcapsules. Prakash et al. [48] also stated that the nanoparticle size with a large surface area gave unique characteristics to the nanomaterials and contributed to their suitability and effectiveness in the food system. These results were within the particle size range reported by Mohammed et al. [28] (488–912 nm), and the PDI reported by Korma et al. [45] (0.22–0.47 nm).

#### 3.2.3. Moisture Content

Moisture content is a critical characteristic of nanocapsules. This characteristic is associated with drying efficiency, water activity, stickiness, flowability, bioactivity, oxidation, and microbial growth [49]. The moisture content of the NCEO ranged from 3.87% to 5.71% (Table 4), which satisfied the requirements for food powders (<6%) [50]. The prepared NCEO-MD had the lowest value, and no significant differences were observed between it and most formulations. By contrast, the NCEO-GA formulation had the highest value. Rodea-González et al. [51] obtained comparable results that ranged from 4.34–5.38% for chia EO microcapsules. However, these results were higher than those found by Fernandes et al. [52], which ranged from 0.88% to 1.76% for ginger EO nanocapsules. By contrast, the results revealed that combining WPI with MD or GA did not affect the moisture content. No significant differences appeared between the NCEO compared with the lowest value obtained in this study. By contrast, the combination of WPI with MD and GA increased the moisture content of the prepared NCEO, but to a lesser extent than the NCEO containing GA individually. However, except for NCEO-GA, all formulations contained less than 5% moisture content, which indicates their storage capability for extended periods [17].

#### 3.2.4. Hygroscopicity

Hygroscopicity is the tendency of powders to absorb moisture. This tendency is a critical quality parameter of encapsulated oils because water absorption during storage can lead to lipid oxidation [53]. Table 4 shows that the hygroscopicity of the NCEO ranged from 12.05% to 15.42%. These values were lower than those obtained by Frascareli et al. [54] for coffee oil nanocapsules (13.83–17.73%) and also lower than rosemary EO nanocapsules (15.87–18.90%), as reported by Fernandes et al. [55]. However, these values were higher than the values of refined kenaf seed oil nanocapsules (7.8–10.1%) achieved by Chew et al. [56]. The results showed that the NCEO-MD/WPI and NCEO-WPI had the lowest hygroscopicity value without significant differences. By contrast, the formulation containing the WPI combination with GA and that of NCEO-MD and then NCEO-GA/MD had the highest hygroscopicity without significant differences. This finding can be attributed to the significant impact of the wall material composition on the hygroscopicity characteristics [21]. Karrar et al. [19] cited that the absorption of moisture by the CHO is attributed to the presence of hydrogen (H_2_) in water components and the availability of hydroxyl groups in the MD and GA.

#### 3.2.5. Solubility

Solubility is a crucial factor in nanocapsules used as additives. Poorly soluble nanocapsules can cause difficulties in processing and economic losses [56]. Table 4 shows that the solubility of the NCEO formulations ranged from 81.87% to 93.85%. The NCEO-MD and NCEO-GA formulations had the highest and lowest solubility values, respectively. These results were higher than the microcapsules of the gurum seed oil results (86.61–90.90%), which were obtained by Karrar et al. [19], and the results for the refined kenaf seed oil nanocapsules (85.6–90.2%), obtained by Chew et al. [56]. Statistically, all the formulations containing WPI did not show any significant differences from the rest. The evidence indicating that the WPI played a role in enhancing the solubility of the formulations has proven that the WPI increases the solubility of powder [21]. Moreover, the WPI and lactose from dairy protein powders have effectively solubilized easily and quickly in the water [21]. Fournaise et al. [57] also reported that the solubility of dairy protein powders linearly varied with the WPI/casein ratio based on the analyses of the reconstituting capability of powder, and is significantly improved by the presence of WPI in the formulations compared with casein.

#### 3.2.6. Wettability

Nanocapsules’ water absorption, or wettability potential, is one of the most critical physical properties associated with powder reconstitution. The wettability time of the NCEO preparations ranged from 108 to 305 s. The NCEO-MD and NCEO-WPI formulations, respectively, had the shortest and longest time for wettability (Table 4). These values were within the range reported by Fernandes et al. [52] on ginger essential oil (91–328 s), and less than those reported by Karrar et al. [19] for gurum seed oil (285–765 s). Furthermore, the wettability was significantly different between the prepared NCEO formulations, except between NCEO-MD/WPI and NCEO-GA/MD/WPI. By contrast, NCEO-MD affected wettability. These results were consistent with those observed by Özbek and Ergönül [43], who reported that the amount of WPI significantly increased the wettability time of the microcapsules. By contrast, the synergistic effect of the MD and GA combination was significantly decreased. Protein–protein interactions induced by the hydrophobic interactions of the protein content on the surface of microparticles, can explain the positive connection between WPI quantity and wettability time [58]. Moreover, the higher hydrophilic nature in MD than WPI can promote the spread of water into the particles, and reduce the wetting time of microcapsules containing CEO [59]. However, the wettability depends on the particle size, density, porosity, and behavior of the amphipathic compounds on the surface [52].

#### 3.2.7. Bulk, Tapped, and Particle Density

Density is an important property in the reconstitution and packaging of nanoencapsulated powders. It is contingent on the degree of inter particulate space or porosity [60]. Powder density is related to storage requirements because high-density powders need smaller storage spaces than low-density powders [52]. The bulk density of the NCEO formulations ranged from 0.26 to 0.36 g/cm^3^. The NCEO-WPI, NCEO-GA/WPI, and NCEO-MD/WPI formulations had the highest bulk density (Table 5). In contrast, the lowest value was achieved by NCEO-GA, NCEO-MD, and NCEO-GA/MD formulations. In general, the bulk density of all the formulations was within the usual range for nanoencapsulated powders [56]. These values matched those obtained by Fernandes et al. [52] (0.27–0.31 g/cm^3^) and mentioned by Chew et al. [56] (0.2–0.3 g/cm^3^). Moreover, the results indicated that the presence of WPI in the mixtures evidently increased and enhanced the bulk density of NCEO powders compared to formulations, either single or blend. However, the powders’ lower bulk density values indicate more air spaces between the particles; therefore, these powders are expected to be more susceptible to oxidative degradation during storage [43].

On the other hand, the tapped density of the NCEO preparations is shown in Table 5 and were ranged from 0.43 to 0.54 g/cm^3^. The NCEO-GA and NCEO-MD formulations had the lowest tapped density, while the NCEO-MD/WPI formulation had the highest value. These values were similar to 0.46–0.53 g/cm^3^, the values obtained by Fernandes et al. [52]. The findings also revealed that the combination of WPI with polysaccharides increased its tapped density, although it did not significantly differ from the formula of NCEO-GA/MD. Chew et al. [56] observed that the tapped density values ranged from 0.4 to 0.6 g/cm^3^ in the microcapsules of refined kenaf seed oil.

In contrast, the particle density of the NCEO ranged from 1.56 to 1.96 g/cm^3^ (Table 5), as the highest value was found in the NCEO-WPI, which differed significantly from all the samples. In contrast, the lowest particle density was obtained by the NCEO-MD formulation without being significantly different from most formulations. These values were higher than those reported by Chew et al. [56] (0.7–1.2 g/cm^3^) and Fernandes et al. [55] (0.97–1.27 g/cm^3^).

#### 3.2.8. Porosity

Porosity is a critical property, especially in powder reconstitution. Porosity indicates the proportion of the volume of pores and the total volume occupied by nanocapsule powders [56]. The porosity of the NCEO ranged from 66.69% to 75.86%. The NCEO-GA/MD/WPI formulation had the lowest porosity. No significant difference (*p* ≤ 0.05) was found from the NCEO-MD/WPI, NCEO-GA/WPI, NCEO-GA/MD, and NCEO-MD formulations. By contrast, the NCEO-GA formulation had the highest porosity (Table 6). These values were similar to 68% to 75% for açai microcapsules [61], and higher than 61.38–68.50% for fingered citron extract microcapsules [29]. However, the slight variations in porosity values can be attributed to the wall material [12].

#### 3.2.9. Cohesiveness and Flowability

CI and HR are two empirically developed factors that determine flow behavior using bulk density and tapped density. CI is used to evaluate free-flowing or compressibility characteristics, whereas HR is used to evaluate powder cohesiveness. Flowability is a critical factor for quality monitoring that can be evaluated through CI and HR [29]. The results in Table 6 show that the CI of NCEO formulations range from 28.57% to 45.45%. The NCEO-WPI formulation has the lowest CI but is not significantly different from the NCEO-GA/WPI formulation. The NCEO-GA/MD formulation has the highest CI but is not significantly different from the NCEO-MD formulation. These values are higher than 27.35–43.59% for the fingered citron extract microcapsules obtained by Mahdi et al. [29], and less than 43.18–53.13% for the pumpkin seed oil microcapsules found by Özbek and Ergönül [43].

In the same context, the HR ranges from 1.40 to 1.83, indicating high powder cohesiveness, as the NCEO-GA/MD formulation has the highest HR without being significantly different from the NCEO-MD formulation. The NCEO-WPI formulation has the lowest HR and is not significantly different from the NCEO-GA/WPI formulation. The results also show that the WPI decreases the HR in the formulations (Table 6). These values are less than 1.7–2.0 for refined kenaf seed oil nanocapsules observed by Chew et al. [56], and they are within the range of 1.38–1.87 for fingered citron extract microcapsules obtained by Mahdi et al. [29].

According to those results, the flowability ranged from poor to awful. The NCEO-WPI and NCEO-GA/WPI formulations had poor flowability, whereas the NCEO-GA/MD, NCEO-GA, and NCEO-MD formulations had awful flowability (Table 6). Although the flowability of the prepared powders was awful, the formulations prepared with or within their WPI composition had a better flowability than the rest of the preparations. This indicates that WPI played a role in improving the flowability of the prepared powders compared to polysaccharides. These results are in agreement with the fingered citron extract microcapsules (poor to awful), obtained by Mahdi et al. [29]. However, they indicate poor flowability by high CI and HR values, typically related to high interparticle friction [62].

#### 3.2.10. Color

Table 7 itemizes the various color properties of NCEO preparations. The results indicated that all formulations had a high lightness, with the L* values varied from 88.69 to 95.36. In contrast, the a* values ranged from −1.00 to 0.42, indicating a slight tendency to redness. The results showed that the values were higher than 0 in the b* parameter varied from 4.18 to 22.64, indicating a significant trend to yellow. Moreover, the ΔE* parameter significantly differed between samples and ranged from 4.35 to 22.23.

On the other hand, the NCEO-MD formula showed a significant decline in the a*, b*, and ΔE* values compared to WPI and GA. Meanwhile, the integration of WPI with MD and GA (NCEO-GA/MD/WPI) presented moderate values in all color parameters. These results are probably due to the different mechanisms of particle development during production. The outcomes were equivalent to those achieved by Khalifa et al. [17]; they attributed the color variation in their samples to different mechanisms of particle development during drying. Furthermore, one of the primary factors is the color of the wall materials [63]. Furthermore, the encapsulated core material plays a vital role in this difference, with chromatic compounds that can change the color [12].

#### 3.2.11. Glass Transition Temperatures

Glass transition temperature (Tg) is the temperature at which the state of a polymeric material changes from a glassy to a rubbery state. Particles with a high Tg are more stable than those with a low Tg, during treatment and storage [42]. The Tg values of the NCEO formulations ranged from 63.84 °C to 79.11 °C. The NCEO-GA/MD/WPI formulation had the highest Tg, followed by the NCEO-GA/MD (78.55 °C), NCEO-GA/WPI (78.06 °C), NCEO-MD/WPI (75.04 °C), NCEO-GA (69.83 °C), and NCEO-MD (65.36 °C) formulations, while the lowest value was found in the NCEO-WPI formulation. These values were within the range of 54.66 °C to 90.38 °C for microcapsules prepared by a mixture of different proteins obtained by Li et al. [64], and close to those reported by Mohammed et al. [28], who found that the Tg ranged from 68.23 °C to 89.21 °C.

Nevertheless, the Tg values of all formulations were above 60 °C, which is considered to be within the safe range for nanocapsule powder quality and stability during storage. Al-Maqtari et al. [12] cited that a higher Tg temperature value than regular storage temperature is vital for maintaining powder consistency and quality throughout storage. Furthermore, the nanocapsules prepared by WPI, MD, and GA, in this study, presented a relatively high Tg temperature. This phenomenon demonstrates that this NCEO-GA/MD/WPI formulation can be potentially used as a wall material in the food industry. According to Karrar et al. [19], at the Tg temperature, the amorphous material transitions from the highly glassy to the rubbery state, due to increased molecular mobility and a decrease in viscosity. Such a transformation can result in structural changes, such as stickiness and a collapse of the product. Thus, the Tg value is affected by various factors, including the chemical structure, moisture content, and molecular weight of materials [28].

#### 3.2.12. Thermogravimetric Analysis

TG and DTG curves provide information on thermal stability and help us to understand the loss or increase in the weight of materials for analysis, due to temperature changes [65]. Moreover, the TGA is a helpful method for verifying the properties of materials treated at high temperatures [40].

**Figure 1:**
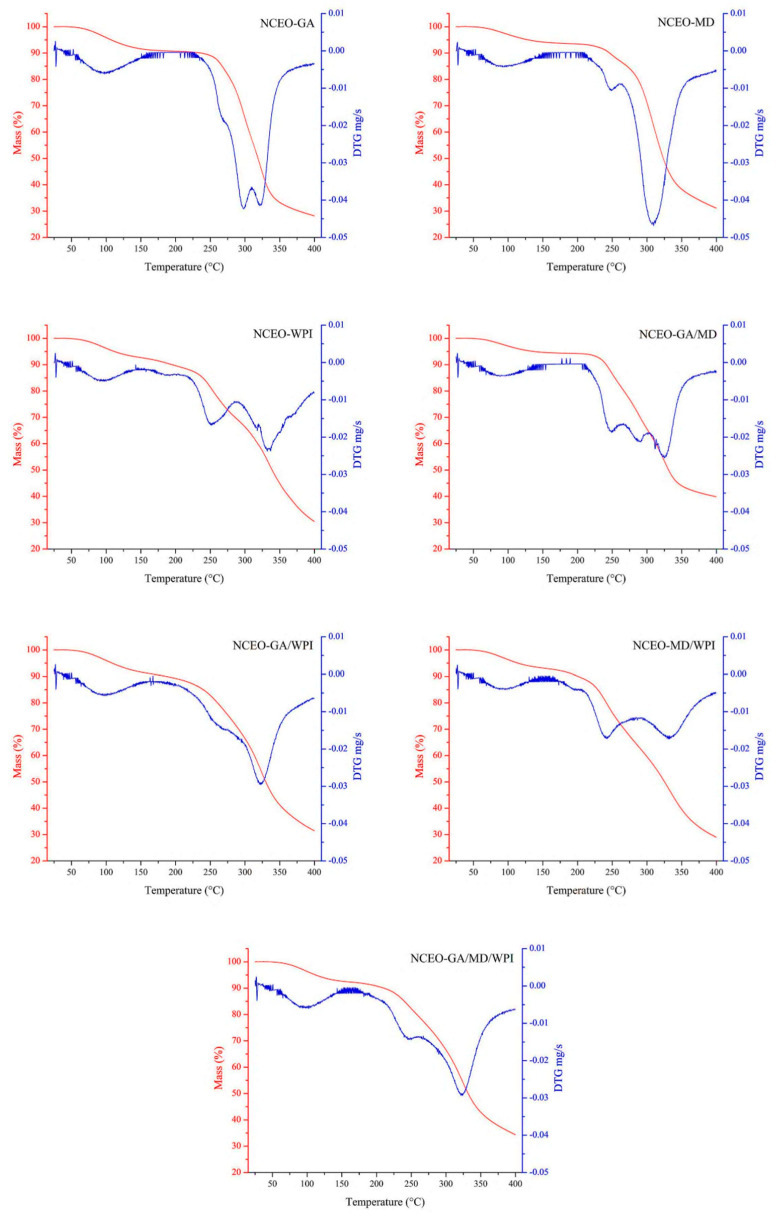
TG and DTG curves of the CEO nanocapsules.

TGA was performed in this study, to estimate the change in the weight of the nanoparticles as a function of temperature. The thermograms of TG and DTG are presented in Figure 1. According to the TG curves, the mass residues ranged from 28.19% to 39.87%. The NCEO-GA/MD formulation had the highest mass residue (39.87%), followed by the NCEO-GA/MD/WPI (34.39%). The lowest mass residue was achieved by NCEO-GA formulation (28.19%) and then NCEO-MD/WPI (28.99%). These values were more than 26.35–37.84% for *Citrus aurantium* EO microcapsules, as indicated by Mohammed et al. [28].

The NCEO-GA formulation, according to the DTG curves, presented four thermal decomposition stages, with temperature peaks (T*_peak_*) at 96.33 °C, 267.67 °C, 297.33 °C, and 321.33 °C. The NCEO-MD formulation had three stages, with T*_peak_* at 90.30 °C, 248.67 °C, and 308.33 °C. The NCEO-WPI formulation presented four stages, with T*_peak_* at 93.00 °C, 251.67 °C, 316.67 °C, and 332.67 °C. Four stages, with T*_peak_* at 90.67 °C, 248.66 °C, 290.00 °C, and 325.33 °C, emerged in the NCEO-GA/MD formulation. The NCEO-GA/WPI formulation presented three stages, with T*_peak_* at 96.33 °C, 271.333 °C, and 322.67 °C. The NCEO-MD/WPI formulation had three stages, with T*_peak_* at 90.66 °C, 241.67 °C, and 332.00 °C. Three stages, with T*_peak_* at 95.33 °C, 247.00 °C, and 322.33 °C, emerged in the NCEO-GA/MD/WPI formulation. The mass loss at 100 °C was 4.13%, 2.95%, 3.79%, 2.94%, 3.99%, 3.68%, and 3.66% for the NCEO-GA, NCEO-MD, NCEO-WPI, NCEO-GA/MD, NCEO-GA/WPI, NCEO-MD/WPI, and NCEO-GA/MD/WPI formulations, respectively. Furthermore, the TGA results showed the difference in the thermal stability of the various NCEO preparations. This variation can be linked to the type of wall materials utilized because all other parameters, such as the emulsion homogenization speed and duration, core-to-wall material ratio, and freeze-drying temperatures, remained constant across all materials [21]. These results demonstrated that the CEO nanocapsules can be used in foods subjected to pasteurization and sterilization temperatures because no remarkable weight loss (mass loss <5%) occurred at 100 °C [65,66].

#### 3.2.13. Crystallinity

XRD analysis is used to estimate whether nanocapsules have a crystalline or amorphous structure. Nanocapsule stability is related to crystallinity, wherein a high relative crystallinity indicates a high stability [49].

**Figure 2:**
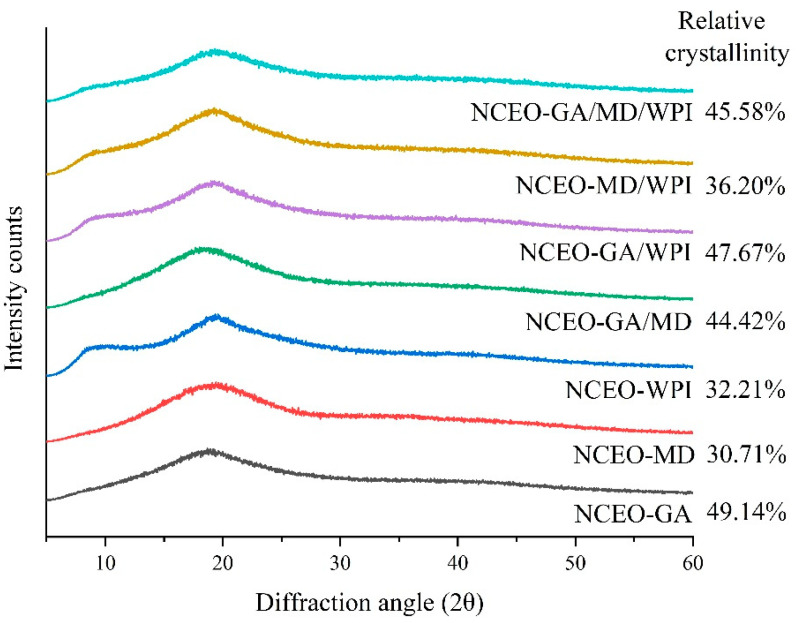
XRD diffraction patterns of the CEO nanocapsules.

The relative crystallinity of the NCEO ranged from 30.71% to 49.14%. The NCEO-GA formulation had the highest relative crystallinity, followed by the NCEO-GA/WPI (47.67%), NCEO-GA/MD/WPI (45.58%), NCEO-GA/MD (44.42%), NCEO-MD/WPI (36.20%), and NCEO-WPI (32.21%) formulations. Meanwhile, the NCEO-MD formulation had the lowest relative crystallinity (Figure 2). These values were higher than those reported by Mohammed et al. [28] (21.76–35.13%), but lower than those reported by Raeisi et al. [46] (42.7–48.8%). Crystallinity influences the rehydration properties of nanocapsules, such as wettability and solubility. Therefore, crystalline capsules are slightly rehydratable and hygroscopic than amorphous ones [13].

#### 3.2.14. Molecular Docking Simulation

**Figure 3:**
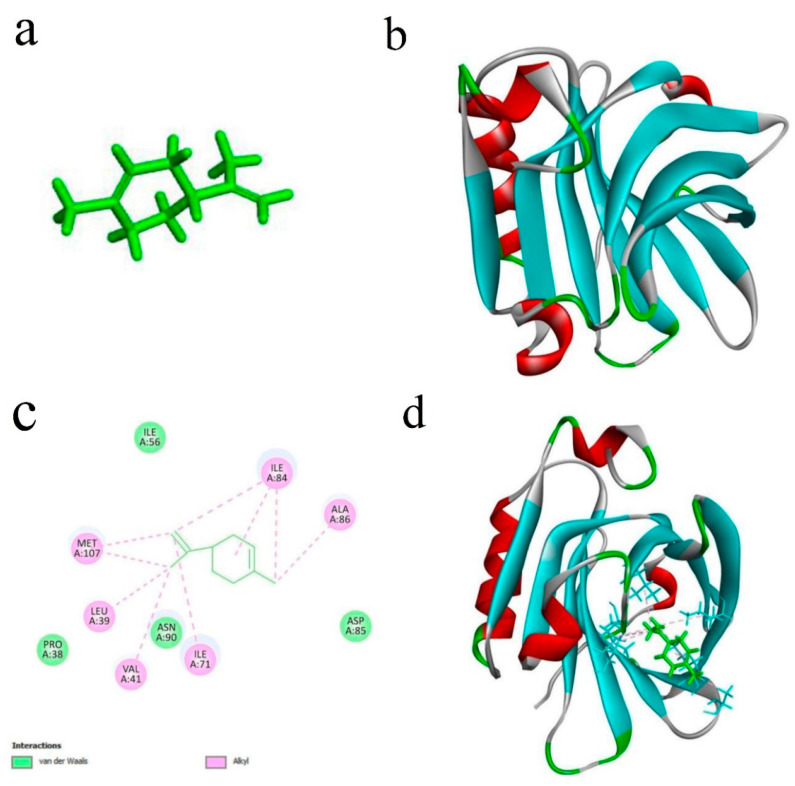
Molecular docking simulation of D-Limonene with β-lactoglobulin. The 3D structure of D-Limonene (**a**), 3D structure of β-lactoglobulin (**b**), 2D diagram of the interactions between D-Limonene with β-lactoglobulin amino acids (**c**), and the 3D diagram of β-lactoglobulin/D-Limonene (**d**).

Computational chemistry can provide the necessary evidence for experimental work, which aids in determining the binding sites and driving forces of interaction [33]. Docking studies are used to prognosticate the favored orientation of one molecule to another, when the two molecules create a stable complex [67]. The major compound of CEO (D-limonene) and the main protein of WPI (β-lactoglobulin) were selected to perform the docking simulation (Figure 3a,b).

Figure 3c shows the participation of 10 amino acid residues in the binding of D-Limonene with β-lactoglobulin. D-Limonene can interact with LEU39 (4.83 Å); VAL41 (4.23 Å); ILE71 (5.09 Å); ILE84 (4.41, 5.15, and 5.25 Å); ALA86 (3.91 Å); and MET107 (4.61 and 4.84 Å) of β-LG via alkyl, in the main-chain positions. Moreover, 4 amino acid residues (PRO38, ILE56, ASP85, and ASN90) were involved in van der Waals forces between D-Limonene with β-lactoglobulin. Moreover, the molecular modeling showed that D-Limonene was bound to β-lactoglobulin in the hydrophobic regions. This result shows that the active groups of the WPI amino acids can intermolecularly interact with D-Limonene (Figure 3d), which is most likely responsible for the stabilization. These results confirm the important role of WPI as a wall material in the CEO encapsulation process.

#### 3.2.15. Morphology of the Nanocapsules

**Figure 4:**
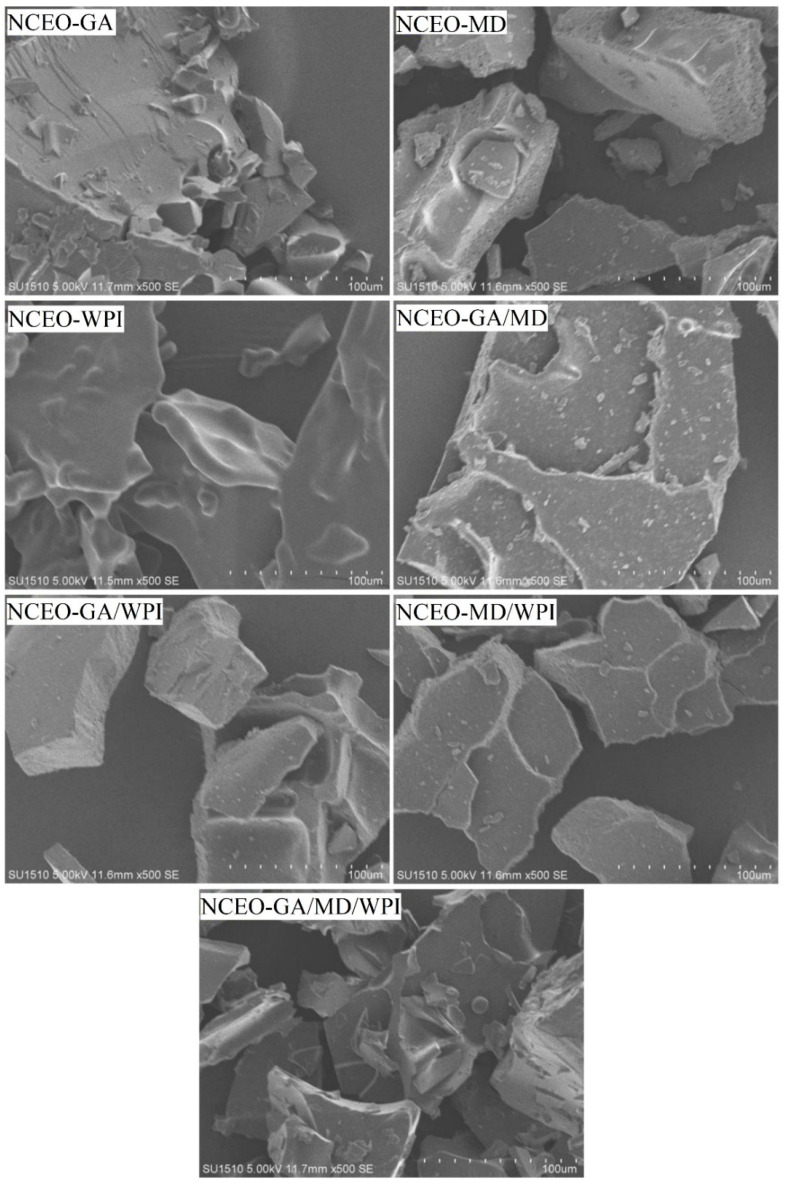
Morphology of the CEO nanocapsules by scanning electron microscopy (SEM).

The SEM was used to observe the external microstructure of CEO nanocapsules, which indicates the stability of the encapsulated oil. The SEM images in Figure 4 showed a marked difference between all the formulations of NCEO. The NCEO-GA formulation demonstrated larger lumps than the other formulations and notably had smooth edges, and the surface was free of cracks or pores.

These findings are in line with those of Ramakrishnan et al. [68], who pinpointed that the surfaces of the capsules containing GA were smoother than those containing other wall materials. By contrast, the NCEO-MD formulation appeared as large sheets with sharp edges and large pores. The NCEO-WPI formulation was entirely different, appearing in the form of thin and curved sheets with a remarkably smooth surface. This result agrees with Yazicioglu et al. [44], who found that the WPI endows the capsules with a smooth surface free of cracks, while MD leads to rough surfaces. This finding can be due to the slow drying rate of WPI and the elasticity provided to the wall systems of the capsules. The combination of GA and MD into the NCEO-GA/MD formulation appeared in the form of sheets in layers with coarse edges, but with smaller pores than the NCEO-MD formulation. The NCEO-GA/WPI formulation appeared in the form of sheets with comparatively rough surfaces and edges that were also free of pores and cracks.

By contrast, the NCEO-MD/WPI formulation was in the form of sheets with several layers and cracks, while nanocapsules appeared in sheets with a surface and edges that were rather smooth and free from cracks and pores when the three wall materials were mixed to form NCEO-GA/MD/WPI. The SEM images were consistent with the encapsulation efficiency and the oxidative stability because the NCEO-MD formulation had the lowest efficiency and stability. This finding can be due to the presence of many large pores, which leads to the leakage of the essential oil from the nanocapsules and their exposure to oxidation, in which the main weakness of the porous structure is the increase in air diffusion into the core material [28].

#### 3.2.16. Stability of the Antioxidant Activity

**Figure 5:**
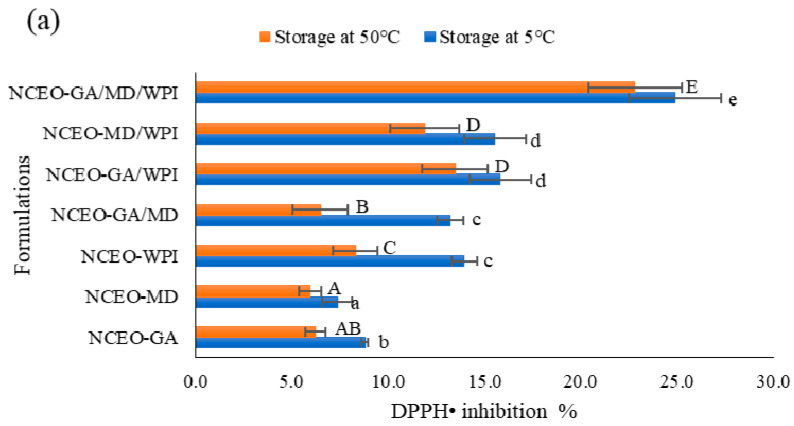

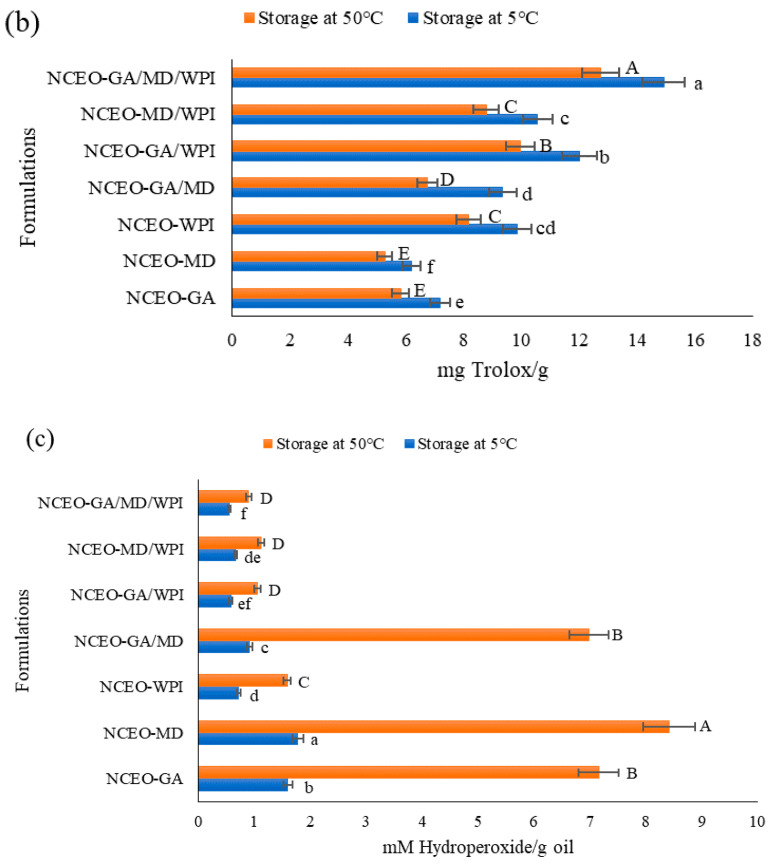
Antioxidant activity stability by DPPH assay (**a**), antioxidant capacity stability by ABTS assay (**b**), and oxidative stability (**c**) of CEO nanocapsules. The results are expressed as the mean ± standard deviation (*n* = 3). Different lower-case letters (a–f) indicate the significant differences (*p* ≤ 0.05) between the stored nanocapsules at 5 °C, and upper-case letters (A–E) indicate the significant differences between the stored nanocapsules at 50 °C.

The antioxidant activity of the stored CEO preparations is presented in Figure 5a. The results showed that the stored formations at 5 °C ranged from 7.36% to 24.88%, while the antioxidant activity of the stored NCEO nanocapsules at 50 °C ranged from 5.96% to 22.80%. Significantly, the NCEO-GA/MD/WPI formulation had the highest antioxidant activity compared with all the formulations, indicating the highest stability. By contrast, the NCEO-MD formulation had the lowest antioxidant activity. Moreno et al. [69] noted that the use of MD in encapsulating grape marc extract did not improve the antioxidant activity compared with WPI. Khalifa et al. [17] also observed that the formulation containing MD had the lowest antioxidant activity among the other preparations, indicating that MD has a limited effect on antioxidant chemical protection.

The results also indicated that the WPI increased the activity of antioxidants in all formulations and stored at 50 °C, compared with MD or/and GA. Except for NCEO-GA/WPI and NCEO-MD/WPI formulations, the difference was significant between all formulations. This finding can be due to the heat-denatured WPI when exposed to high temperatures [18]. This phenomenon produces free sulphydryl groups, which have free radical scavenging properties [70].

The rate of antioxidant activity degradation at 5 °C was 2.82, 3.38, 1.78, 1.88, 1.57, and 1.60 times for NCEO-GA, NCEO-MD, NCEO-WPI, NCEO-GA/MD, NCEO-GA/WPI, and NCEO-MD/WPI formulations, respectively, compared with NCEO-GA/MD/WPI formulation. At 50 °C, the rate of antioxidant activity degradation for the same formulations was 3.67, 3.82, 2.75, 3.52, 1.69, and 1.92 times, compared with NCEO-GA/MD/WPI formulation. These results are compatible with the encapsulation efficiency because the high-efficiency CEO nanocapsules have high stability in antioxidant activity.

#### 3.2.17. Stability of the Antioxidant Capacity

As observed in Figure 5b, the antioxidant capacity stability of NCEO formulations was dependent on the wall material and varied from 6.21 to 14.90 mg Trolox/g for formulations stored at 5 °C, and from 5.26 to 12.74 mg Trolox/g between the NCEO preparations stored at 50 °C. The NCEO-GA/MD/WPI formulation had the highest value (14.90 and 12.74) at 5 °C and 50 °C, respectively, followed by NCEO-GA/WPI (12.00 and 9.94), NCEO-MD/WPI (10.56 and 8.78), and NCEO-WPI (9.83 and 8.16). Simultaneously, the lowest antioxidant capacity was observed in NCEO-MD (6.21 and 5.26) and NCEO-GA (7.17 and 5.82) at 5 °C and 50 °C, respectively, without significant differences at 50 °C. These results proved that adding WPI to the formulations raised the efficiency of the antioxidant capacity compared with MD and GA.

This result was confirmed by Moreno et al. [69], who found that using MD as a wall material had no effect on the antioxidant of grape marc extract when compared to WPI. Similar findings were obtained by Khalifa et al. [17], who found that the formulation containing MD had a lower antioxidant activity and capacity than the other formulations, representing the limited effect of MD on the protection of antioxidants compounds.

By contrast, the rate of antioxidant capacity degradation for the stored NCEO formulations at 5 °C was 2.08, 2.40, 1.52, 1.59, 1.24, and 1.41 times for NCEO-GA, NCEO-MD, NCEO-WPI, NCEO-GA/MD, NCEO-GA/WPI, and NCEO-MD/WPI formulations, respectively, compared with the NCEO-GA/MD/WPI formulation. Meanwhile, this rate was 2.19, 2.42, 1.56, 1.89, 1.28, and 1.45 times at 50 °C. The results indicate that mixtures of wall materials, especially the triple mixture of GA, MD, and WP, yielded better results than single wall materials, which contributed to improving the encapsulation efficiency and enhancing the stability of the CEO nanocapsules during storage. Similar results were detected by Khalifa et al. [17], who observed that the highest stability for antioxidant activity and capacity was observed in the formulation containing GA/MD/WPI. Furthermore, they showed that the preparations with WP and/or GA alone or together as wall materials had the highest antioxidant activity and capacity because of their electron-donating capability.

#### 3.2.18. Oxidative Stability

The oxidative stability of NCEO preparations was assessed using the peroxide value (POV), which measures the primary oxidation of the oil. The POV of NCEO formulations with various wall materials was measured after storage at 5 °C and 50 °C, and the results are presented in Figure 5c. These results showed that the wall material composition substantially impacted oxidative stability because the POV of the stored NCEO preparations at 5 °C ranged from 0.55 mM to 1.78 mM hydroperoxide/g oil. By contrast, the NCEO formulations stored at 50 °C, ranged from 0.90 mM to 8.42 mM hydroperoxide/g oil. The NCEO-GA/MD/WPI formulation had the lowest POV without significantly different changes compared with the NCEO-GA/WPI formulation. The results also revealed that the use of WPI within the compositions of wall materials contributes to improving oxidative stability. These findings are in agreement with those Lekshmi et al. [21], who found that MD-WPI-based formulations had the best oxidative stability, followed by GA-based. This finding can be attributed to the high oxidative stability of MD-WPI, due to the combined properties of the wall material. Another study also demonstrated that a combination of WPI with polysaccharides provided reasonable protection against lipid oxidation [71].

By contrast, the findings revealed that temperatures had a role in oxidative stability in NCEO preparations. Oxidative degradation was lower in stored NCEO formulations at 5 °C, compared with those stored at 50 °C. The POV of NCEO-GA, NCEO-MD, NCEO-WPI, NCEO-GA/MD, NCEO-GA/WPI, and NCEO-MD/WPI formulations at 5 °C respectively increased by 2.89, 3.22, 1.30, 1.66, 1.05, and 1.21 times, compared with the NCEO-GA/MD/WPI formulation, while that at 50 °C respectively increased by 7.98, 9.39, 1.77, 7.79, 1.17, and 1.25 times. This result is in line with Al-Maqtari et al. [12], who showed that temperature played a role in rising oxidative stability in microcapsules, in which the oxidative stability was higher in microcapsules held at 4 °C compared to those stored at 25 °C. Moreover, the authors noted that combining proteins with polysaccharides improved and delayed oxidation degradation over time compared with GA, individually. These results were consistent with encapsulation efficiency because formulations with low efficiency have a low stability. Moreover, the results proved that combining different wall materials leads to a better EO encapsulation than those singly used. Overall, the nanoencapsulation of CEO by GA/MD/WPI enhanced the oxidative stability of the core materials, with the maximum improvement observed when WPI was used within the wall materials.

## 4. Conclusions

This study aimed to enhance the encapsulation efficiency of CEO using the WPI with a combination of GA and MD, as wall materials. Also, the physicochemical, structural, thermal, morphological properties, antioxidant activity, and oxidative stability were measured. Moreover, the interaction between CEO and WPI was confirmed by molecular docking. The NCEO-WPI formulation had better results compared with the NCEO formulations that used polysaccharides, individually. By contrast, the mixed formulations containing WPI, especially the NCEO-GA/MD/WPI formulation, outperformed the other mixed formulations. This study revealed that the use of WPI within the compositions of wall materials, especially with MD and GA, improves the physicochemical and stability properties of the nanoencapsulation of CEO, and can be applied to encapsulate other EOs. Conducting further studies on using GA/MA/WPI mixtures to encapsulate other oils is recommended due to the proven high encapsulation efficiency.

## Figures and Tables

**Table 1:** Formulation of the aqueous phase of *Citrus* *reticulata* essential oil nanoemulsions.

Formulations Code	Gum Arabic (g)	Maltodextrin (g)	Whey Protein Isolate (g)	Water (g)
NCEO-GA	24	-	-	68
NCEO-MD	-	24	-	68
NCEO-WPI	-	-	24	68
NCEO-GA/MD	12	12	-	68
NCEO-GA/WPI	12	-	12	68
NCEO-MD/WPI	-	12	12	68
NCEO-GA/MD/WPI	8	8	8	68

GA formulation (NCEO-GA); MD formulation (NCEO-MD); WPI formulation (NCEO-WPI); GA/MD formulation (NCEO-GA/MD); GA/WPI formulation (NCEO-GA/WPI); MD/WPI formulation (NCEO-MD/WPI); GA/MD/WPI formulation (NCEO-GA/MD/WPI); and - (0 g).

**Table 2:** MSD, PDI, and ζ-potential of fresh emulsions.

Emulsions’ Code	MSD (nm)	PDI	Zeta-Potential (mV)
GA-based	674.18 ± 10.07 a	0.26 ± 0.03 a	−58.08 ± 0.28 b
MD-based	628.59 ± 13.5 b	0.23 ± 0.03 ab	−57.48 ± 0.24 a
WPI-based	357.86 ± 12.39 e	0.17 ± 0.05 c	−57.64 ± 0.33 ab
GA/MD-based	578.58 ± 8.02 c	0.25 ± 0.03 ab	−57.66 ± 0.26 ab
GA/WPI-based	527.12 ± 8.25 d	0.23 ± 0.04 ab	−57.69 ± 0.28 ab
MD/WPI-based	372.18 ± 8.29 e	0.23 ± 0.04 ab	−57.75 ± 0.26 ab
GA/MD/WPI-based	529.64 ± 15.51 d	0.20 ± 0.04 bc	−57.75 ± 0.28 ab

The data are shown as the mean ± SD (*n* = 3). In the same column, the different letters indicate that the data is significantly different (*p ≤* 0.05).

**Table 3:** Encapsulation efficiency, particle size, and polydispersity index (PDI) of CEO nanocapsules.

Formulations	Encapsulation Efficiency (%)	Particle Size (nm)	PDI
NCEO-GA	37.17 ± 3.16 d	782.09 ± 58.66 a	0.47 ± 0.04 d
NCEO-MD	36.29 ± 2.90 d	695.49 ± 52.16 abc	0.41 ± 0.03 c
NCEO-WPI	65.55 ± 5.90 c	427.35 ± 32.05 d	0.26 ± 0.02 a
NCEO-GA/MD	43.04 ± 3.23 d	738.51 ± 55.39 ab	0.36 ± 0.03 bc
NCEO-GA/WPI	78.22 ± 6.65 b	641.79 ± 48.13 c	0.32 ± 0.02 b
NCEO-MD/WPI	69.03 ± 4.83 c	430.35 ± 32.28 d	0.27 ± 0.02 a
NCEO-GA/MD/WPI	92.08 ± 6.45 a	674.95 ± 50.62 bc	0.38 ± 0.03 c

The data are shown as the mean ± SD (*n* = 3). In the same column, the different letters indicate that the data is significantly different (*p ≤* 0.05).

**Table 4:** Moisture, hygroscopicity, solubility, and wettability of CEO nanocapsules.

Formulations	Moisture (%)	Hygroscopicity (%)	Solubility (%)	Wettability (s)
NCEO-GA	5.71 ± 0.37 a	12.90 ± 0.77 cd	81.87 ± 3.68 b	247.3 ± 17.5 c
NCEO-MD	3.87 ± 0.29 c	14.63 ± 0.95 ab	93.58 ± 4.68 a	108.0 ± 10.0 f
NCEO-WPI	4.31 ± 0.26 c	12.21 ± 0.61 d	89.61 ± 4.93 ab	305.3 ± 25.0 a
NCEO-GA/MD	3.95 ± 0.26 c	13.94 ± 0.77 abc	84.12 ± 3.79 b	172.0 ± 9.20 e
NCEO-GA/WPI	4.12 ± 0.31 c	15.42 ± 0.93 a	83.25 ± 4.16 b	286.0 ± 10.0 b
NCEO-MD/WPI	3.93 ± 0.28 c	12.05 ± 0.72 d	86.51 ± 4.33 ab	223.0 ± 14.4 d
NCEO-GA/MD/WPI	4.98 ± 0.35 b	13.84 ± 0.90 bc	87.55 ± 4.82 ab	242.3 ± 0.39 cd

The data are shown as the mean ± SD (*n* = 3). In the same column, the different letters indicate that the data is significantly different (*p ≤* 0.05).

**Table 5:** Bulk, tapped, and particle density of CEO nanocapsules.

Formulations	Bulk Density	Tapped Density	Particle Density
NCEO-GA	0.26 ± 0.03 c	0.43 ± 0.04 c	1.79 ± 0.06 b
NCEO-MD	0.26 ± 0.03 c	0.46 ± 0.04 bc	1.56 ± 0.08 c
NCEO-WPI	0.36 ± 0.04 a	0.50 ± 0.04 ab	1.96 ± 0.09 a
NCEO-GA/MD	0.28 ± 0.03 c	0.52 ± 0.04 a	1.67 ± 0.08 bc
NCEO-GA/WPI	0.36 ± 0.04 a	0.52 ± 0.05 a	1.61 ± 0.06 c
NCEO-MD/WPI	0.36 ± 0.03 a	0.54 ± 0.05 a	1.67 ± 0.09 bc
NCEO-GA/MD/WPI	0.32 ± 0.04 b	0.52 ± 0.04 a	1.67 ± 0.06 bc

The data are shown as the mean ± SD (*n* = 3). In the same column, the different letters indicate that the data is significantly different (*p* ≤ 0.05).

**Table 6:** Porosity, Carr’s index, Hausner ratio, and flowability of CEO nanocapsules.

Formulations	Porosity (%)	Carr’s Index (%)	Hausner Ratio	Flowability
NCEO-GA	75.86 ± 3.79 a	38.95 ± 1.95 b	1.64 ± 0.08 bc	Awful
NCEO-MD	72.22 ± 3.97 abc	43.75 ± 2.41 a	1.78 ± 0.10 ab	Awful
NCEO-WPI	74.45 ± 3.35 ab	28.57 ± 1.29 d	1.40 ± 0.06 d	Poor
NCEO-GA/MD	68.75 ± 3.44 bc	45.45 ± 2.27 a	1.83 ± 0.09 a	Awful
NCEO-GA/WPI	68.00 ± 3.74 bc	30.00 ± 1.65 d	1.43 ± 0.08 d	Poor
NCEO-MD/WPI	67.39 ± 3.03 c	34.29 ± 1.54 c	1.52 ± 0.07 cd	Very poor
NCEO-GA/MD/WPI	66.69 ± 3.33 c	37.66 ± 1.88 b	1.60 ± 0.08 c	Very poor

The data are shown as the mean ± SD (*n* = 3). In the same column, the different letters indicate that the data is significantly different (*p ≤* 0.05).

**Table 7:** Color parameters of CEO nanocapsules.

Formulations	L*	a*	b*	ΔE*
NCEO-GA	92.31 ± 0.20 d	0.42 ± 0.01 e	8.92 ± 0.12 c	8.26 ± 0.12 c
NCEO-MD	95.36 ± 0.20 f	−1.00 ± 0.11 a	4.18 ± 0.29 a	4.35 ± 0.21 a
NCEO-WPI	88.69 ± 0.09 a	−0.13 ± 0.02 d	22.64 ± 0.19 f	22.23 ± 0.17 f
NCEO-GA/MD	93.29 ± 0.08 e	−0.16 ± 0.01 d	6.90 ± 0.05 b	6.20 ± 0.04 b
NCEO-GA/WPI	89.83 ± 0.18 b	0.41 ± 0.04 e	16.48 ± 0.71 e	16.01 ± 0.73 e
NCEO-MD/WPI	91.61 ± 0.25 c	−0.75 ± 0.04 b	16.58 ± 0.57 e	15.83 ± 0.59 e
NCEO-GA/MD/WPI	93.52 ± 0.07 e	−0.35 ± 0.08 c	11.39 ± 0.22 d	10.66 ± 0.21 d

The data are shown as the mean ± SD (*n* = 3). In the same column, the different letters indicate that the data is significantly different (*p* ≤ 0.05). Lightness (L*), redness (a*), yellowness (b*), and the total color difference (ΔE*).

## Data Availability

Not applicable.
